# Effects of Additional Intra-aortic Balloon Counter-Pulsation Therapy to Cardiogenic Shock Patients Supported by Extra-corporeal Membranous Oxygenation

**DOI:** 10.1038/srep23838

**Published:** 2016-04-01

**Authors:** Lian-Yu Lin, Che-Wei Liao, Chih-Hsien Wang, Nai-Hsin Chi, Hsi-Yu Yu, Nai-Kuan Chou, Juey-Jen Hwang, Jiunn-Lee Lin, Fu-Tien Chiang, Yih-Sharng Chen

**Affiliations:** 1 Division of Cardiology, Department of Internal Medicine, National Taiwan University College of Medicine and Hospital, Taipei, Taiwan; 2 Division of Cardiology, Department of Internal Medicine, National Taiwan University Hospital, Hsin-Chu Branch, Hsin-Chu City, Taiwan; 3 Division of Cardiology, Department of Surgery, National Taiwan University College of Medicine and Hospital, Taipei, Taiwan; 4 Division of Cardiology, Department of Surgery, National Taiwan University Hospital, Hsin-Chu Branch, Hsin-Chu City, Taiwan

## Abstract

Extra-corporeal membranous oxygenation (ECMO) has been applied in patients with cardiopulmonary failure. One critical drawback of peripheral ECMO is an increase in left ventricular (LV) afterload which could be counterbalanced by the combination of intra-aortic balloon counter-pulsation (IABP) therapy. We hypothesized that an add-on therapy with IABP could improve outcomes in patients receiving ECMO support. We included patients (>18 years old) from 2002 to 2013 requiring ECMO support due to cardiogenic shock in a medical center. A total of 529 patients (227 ECMO alone and 302 combined IABP plus ECMO) were included. The mortality rates at 2 weeks (48.5 vs. 47.7%) after ECMO implantation were not different between the two groups (ECMO vs. combined group). After adjustment for propensity score and potential confounders, the odds ratios of outcomes within 14 days (combined group vs. ECMO) for poor LV systolic function, high preload, multi-organ failure and mortality were not different. The results remained similar for subgroup analysis. Compared with ECMO alone, combined IABP and ECMO treatment did not improve outcomes in patients with circulatory failure.

Evolved from the 1970s, extra-corporeal membranous oxygenation (ECMO) has been widely used to support patients with respiratory or circulatory failure^1^ and a successful bridge for severe heart failure patients to ventricular assistant device (VAD) or transplantation due to various etiologies such as myocardial infarction^2^,^3^, dilated cardiomyopathy^4^, myocarditis^5^, cardiac surgery complications^6^, or cardiac arrest^7^. There are several forms of ECMO, the most common one for cardiac support is the veno-arterial (V-A). In critical condition, V-A ECMO usually is delivered peripherally. The blood is drained from a venous cannula usually placed in the femoral vein and retrogradely perfuses vital organs through cannulation in the femoral artery. Although peripheral V-A ECMO can reduce LV preload, it can in turn lead to an increase in ventricular wall tension due to retrograde flow.

Intra-aortic balloon counter-pulsation (IABP), another standardized mechanical circulatory support, is considered to improve coronary perfusion, increase LV stroke volume, decrease LV wall stress and myocardial oxygen demand. Some studies have demonstrated that combing IABP in an ECMO-supported patient for cardiogenic shock seems to be an effective mechanical circulatory support modality^8^,^9^ and might potentially prevent the ECMO associated lung edema by reducing pulmonary artery pressure with acceptable complication rate^10^. However, these reports are small series and lack of control.

In this study, we planned to investigate whether the combination therapy with IABP and ECMO is superior to ECMO alone in improving outcomes in critically ill patients requiring V-A ECMO rescue.

## Results

### Basic characteristics

**Figure 1:**
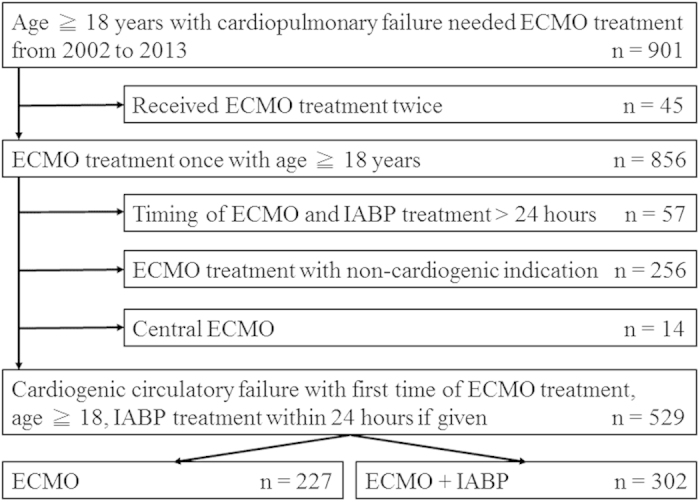
The patient selection algorithm. ECMO: extracorporeal membrane oxygenation, IABP: intra-aortic balloon pumping.

From Jan. 1, 2002 to Dec. 31, 2013, a total of 901 adult patients had received circulatory support by ECMO at our hospital. For simplicity, we excluded 45 patients who had received multiple ECMO treatment due to condition deterioration after weaning ECMO. Fifty-seven patients were also excluded since the timings of implanting ECMO and IABP were more than 24 hours and died in 24 hours in combined treatment group. Among the remaining 799 patients, we included only 529 subjects who received peripheral V-A ECMO treatment due to cardiogenic circulatory failure. The patient selection algorithm was shown in Fig. 1.

The basic characteristics were summarized in Table 1. As shown in Table 1, in the study population, 302 patients received combined ECMO and IABP treatment while 227 patients received ECMO support only. Patients in combined group were older (56.8 ± 13.4 vs. 52.8 ± 17.2 years, *p* = 0.004), had less female gender (20.5 vs. 30.0%, *p* = 0.014), higher body mass index (25.1 ± 3.9 vs. 23.9 ± 4.3 kg/m^2^, *p* = 0.001) and higher prevalence of hypertension (39.4 vs. 29.1%, *p* = 0.017), diabetes mellitus (36.8 vs. 26.4%, *p* = 0.015). More patients in combined group were smoker (28.5 vs. 20.4%, *p* = 0.034). For peri-ECMO period procedure, more patients in combined group received cardio-pulmonary resuscitation (38.1 vs. 29.1, *p* = 0.033), coronary artery bypass graft (38.4 vs. 13.2%, *p* < 0.001) while more patients in ECMO alone group received valvular surgery (11.9 vs. 4.0%, *p* = 0.001) and other surgery (7.0 vs. 3.0%, *p* = 0.038). For etiologies leading to ECMO treatment, more patients in combined group were due to acute coronary syndrome (58.9 vs. 25.6%) and more patients in ECMO alone group were due to cardiomyopathy (27.3 vs. 17.2%), post-cardiotomy (31.7 vs. 12.9%) and acute myocarditis (15.4 vs. 10.9%). The common ECMO set-up sites were intensive care unit (41.6 vs. 28.8%), emergency room (12.4 vs. 25.8%) and other hospital (15.0 vs. 20.5%) for ECMO alone and combined groups respectively.

### Patients’ clinical parameters

The clinical parameters after ECMO implantation were listed in Table 2. Patients in combined group had higher systolic blood pressure (SBP) (107.6 [93.3–120.5] vs. 99.5 [86.3–113.4] mmHg, *p* < 0.001) and lower diastolic blood pressure (DBP) (57.4. [50.3–64.8] vs. 65.1 [54.4–73.1] mmHg, p < 0.001). The other parameters including serum lactic level, daily urine output, arterial blood gas, heart rate (HR), central venous pressure (CVP), inotropic equivalent (IE), and left ventricular ejection fraction (LVEF) were not different between the two groups.

### Patients’ outcomes

The outcomes of the patients were summarized in Table 3. The mortality rate at 2 weeks (48.5 vs. 47.7%, *p* = 0.861 for ECMO alone and combined treatment respectively) was not different between the two groups. The most common etiology of mortality were multi-organ failure (92.2% and 94.2% for ECMO alone and combined treatment respectively). As expected, more patients in combined group received limb fasciotomy operation due to vascular complications (2.6 vs. 0.0%, *p* = 0.012). The other outcomes were not different between the two groups.

The odds ratios for adding IABP support as a determinant for outcomes within 2 weeks were shown in Table 4. After adjustment for potential confounders, the odds ratios of combined group vs. ECMO were 0.730 (95% CI: 0.473–1.126, *p* = 0.154) for poor LV systolic function (LVEF ≦ 35%), 0.775 (95% CI: 0.457–1.313, *p* = 0.343) for high preload (CVP), 1.360 (95% CI: 0.810–2.282, *p* = 0.212) for multi-organ failure, 1.008 (95% CI: 0.666–1.525, *p* = 0.955) for mortality at 2 weeks respectively. After propensity score adjustment, the result remained similar.

Subgroup analysis stratified by etiology of circulatory failure was also demonstrated in Table 4. For patients with acute coronary syndrome, cardiomyopathy and myocarditis, the results were similar to all study group after propensity adjustment. For post-cardiotomy patients, the 2 week-mortality was significantly higher in subjects with combined treatment (odds ratio: 8.398 [95% CI: 1.916–36.805], *p* = 0.005). The result remained similar after propensity score adjustment.

## Discussion

To our knowledge, this study is the largest series comparing the outcomes of combined IAPB plus ECMO with the ECMO alone therapy in critical ill patients. Our results showed that an add-on IABP to ECMO treatment has no additional survival benefits.

Developed in the 1970s’, ECMO has now been applied to a wide variety of critical conditions. Studies have demonstrated that it has beneficial effects in patients with refractory respiratory failure such as acute respiratory distress syndrome^11^, hypercapnic respiratory failure^12^ and could work as a bridging therapy to lung transplantation^13^ or allograft failure^14^. In addition to it’s success in respiratory failure, more and more reports have focused on it’s ability in circulatory mechanical support. Although only observational studies have been reported, ECMO has revealed its potential survival benefit in many critical conditions such as myocardial infarction –related cardiogenic shock^3^, refractory heart failure caused by dilated cardiomyopathy^4^ or acute myocarditis^5^, post-cardiac surgery complications^6^ and cardiopulmonary resuscitation in cardiac arrest patients^7^.

Despite its capability of hemodynamic support and reducing LV pre-load, ECMO would also increase the LV afterload, distend the LV and increase wall stress and thus cause an increase in myocardial oxygen demand and sub-endocardial ischemia that could impede myocardium recovery^9^,^15^. Several treatment techniques have been proposed to avoid these disadvantages such as trans-septal left atrial drainage^16^, Impella assist device^17^ and IABP^9^. IABP has been widely used in patients with cardiogenic shock for decades. Previous studies have shown its hemodynamic benefits in LV after-load reduction and coronary perfusion augmentation^18^. As an adjuvant mechanical support to ECMO, IABP could not only restore the pulsatility of the LV pressure output but also reduce LV end-diastolic diameter and pulmonary artery-occlusion pressure^19^. Although, the mortality benefit conferred by IABP counterpulsation in myocardial infarction patients treated with fibrinolytics is evident^20^,^21^,^22^, but when it comes to percutaneous coronary intervention, study results have been controversial. Recently, a large prospective, randomized, multicenter trial showed that there was no significant difference in the all-cause mortality nor was there a significant difference in major bleeding, peripheral ischemic complications in myocardial infarction-related cardiogenic shocks treated with or without IABP^23^. Our result may also highlight and demonstrate that the effect of IABP does not provide an additional benefit in survival, which was similar in IABP-SHOCK-II trial^23^.

In contrast to several previous reports showing that the combined IABP and ECMO treatment might potentially improve patients’ outcomes^8^,^9^, we found that the combined therapy not only could not improve survival but also could not prevent the development of multi-organ failure. We believed that the lack of control patients and small series in those studies are two main reasons leading to this discrepancy. Since most patients in this study died from multi-organ failure, we hypothesized that the main reason that the combined therapy could not improve survival is its disability to avoid or reverse the complex processes leading to multi-organ failure. Study has found that the introduction of IABP could not reduce the serum lactate level in myocardial infarction patients complicated with cardiogenic shock^23^. Similar result also has been reported in combined therapy as our study^19^. This indicates that IABP treatment alone or in combination with ECMO could not improve micro-circulation, tissue perfusion and thereby prevents organ failure. Notably, in subgroup analysis, post-cardiotomy patients carried a higher risk of mortality in combined treatment group. It is possible that these patients received combined treatment due to their more critical conditions.

One of the major concerns of combined therapy is limb ischemia since both femoral arteries should be cannulated. Our data pointed out that the implantation of IABP would not increase the risk of vascular compliacions, indicating that most of the vascular complications are from ECMO. It is probably that the size of ECMO catheter is more bulky than that of IABP. In this study, nearly half of our patients received the insertion of reperfusion catheter together with ECMO therapy but still there were a small number of cases progressing to more severe ischemia requiring fasciotomy or resulting in gangreneous change of distal extremities.

## Conclusions

In conclusion, compared with ECMO alone, combined IABP and ECMO treatment did not improve survival or prevent multi-organ failure in cardiopulmonary compromised patients.

### Study limitations

This study had several limitations. First, this is an observational cohort study. Second, notwithstanding the propensity score matching has been proved an effective method to balance the covariates between two treatment groups, a large scale randomized-control trial is still needed for a comprehensive evaluation of the effects adding IABP to ECMO. Finally, there was no standard protocol of the timing to initiate IABP or ECMO in this study. It depended on the primary care doctor’s judgment. The different timing of initiation might affect the final outcomes.

## Methods

### Ethics Statement

The research was approved by the institutional review board of the National Taiwan University Hospital Ethics Committee. The study was conducted in accordance with the approved guidelines. Because this was a retrospective observational study, the institutional review board of the National Taiwan University Hospital Ethics Committee (No. 201404079 RIN) waived informed consent.

### Patient populations

The ECMO team consists of cardiac surgeons, intensivists, technicians and multidisciplinary specialists, forming an around-the-clock comprehensive medical care network. ECMO was considered as the treatment of choice in circulatory collapse for mechanical support, either emergently or urgently, in our institute. The criteria of V-A ECMO included those under cardiopulmonary resuscitation, or cardiogenic shock with multiple inotropic support over 35 μg/kg/min inotropic equivalent (IE, = dopamine + dobutamine + (epinephrine + norepinephrine + isoproterenol) × 100 + milrinone × 15) and persistent organs hypoperfusion. The cases requiring urgent ventricular assist device implantation was excluded if they di not received ECMO rescue.

In the current study, we included all adult patients (age ≥ 18 years old) from 2002 to 2013 whether initially admitted to our center or referred from other hospitals with compromised cardiopulmonary system requiring ECMO support. We choice this period since detailed medical information was prospectively collected and stored in an on-line data bank during this period.

In our hospital, whether IABP treatment should be routinely given to patients receiving ECMO support is a debating issue. Some physicians believed that IABP could lower the afterload in patients receiving ECMO while others preferred ECMO monotherapy in order to prevent vascular complications. Around half of the patients received ECMO alone treatment, which offered us a chance to compare the outcomes between patients with combined IABP-ECMO and ECMO alone treatments.

### Extra-corporeal membranous oxygenation technique

In our center, cardiovascular surgical team evaluated every patient to judge whether the ECMO was indicated according to inclusion criteria mentioned above. The femoral vein and/or artery were exposed in a cut-down wound and cannulated via puncture method with CARMEDA cannula (Medtronic Inc., Anaheim, CA). The circuit had heparin-bonded CARMEDA bioactive surface (Medtronic Inc., Anaheim, CA) and was primed by a saline-diluted heparin (2 units/mL). It was connected to an Affinity oxygenator and driven by a Bio-Pump centrifugal blood pump (Medtronic Inc., Anaheim, CA) or Rotaflow (Maquet, Germany). An antegrade perfusion catheter was administered to prevent distal extremity ischemia if necessary. Heparin was continuously infused to keep activated clotting time (ACT) over 220 seconds. Experienced perfusionists or technicians would examine the system daily to maintain adequate flow and ACT, and check for clot formation and oxygenator dysfunction.

### Intra-aortic balloon counter-pulsation technique

The primary care cardiovascular physician or surgeon judged the decision of IABP insertion. A 30 or 40 mL IABP balloon, size judged according to the patient’s height, was inserted through a femoral sheath and with the tip located near the second rib. The support was initiated at a 1:1 inflation-deflation to cardiac cycle ratio, either by electrocardiographic or blood pressure wave form triggering. The removal of the IABP was also judged by the attending physician. Usually, the IABP was removed when the systolic blood pressure remained above 100 mmHg without inotropic agents. In general, IABP would be removed after the ECMO could be weaned off successfully for those with combined IABP and ECMO support.

### Outcomes

The primary outcome was all-cause mortality within 2 weeks. Other outcomes were organ failure (brain, lung, heart, liver, kidney, gastro-intestine) and vascular complications within 14 days. LVEF, LV preload (CVP) in 1 week were also used as outcomes to measure the short-term effect of IABP on LV systolic function and LV preload. There was no worldwide-accepted definition of organ failure. In our critical care center, the definition of organ failure was defined as followed. For brain failure, we defined it as a consciousness change (Glasgow coma scale ≦ 7 after discontinuing sedative and muscle relaxing agents) plus a brain image or EEG study showing hypoxic encephalopathy. If the patients was intubated and received ventilator support, the definition was eye plus motor response of Glasgow coma scale ≦ 6. The definition of heart failure was failure of weaning ECMO or unstable blood pressure (defined as SBP < 90 mmHg or drop of SBP for more than 30 mmHg) plus impaired LVEF (<40%) requiring high dose of catecholamine support with an IE ≥ 20 μg/kg/min. Lung failure was defined as an arterial oxygen saturation < 85% under adequate ventilation support or a ratio between partial pressure of oxygen in arterial blood (PaO2) and fraction of inspired oxygen (FiO2) < 60 or an oxygen index > 30^19^. Liver failure was defined as total bilirubin over 15 mg/dL or elevated liver enzymes (alanine transaminase or aspartate transaminase) over 10 times of normal values. Renal failure was defined as a new-onset kidney disease requiring renal replacement therapy. Gastro-intestinal failure was defined as failure in enteral feeding or massive gastro-intestinal bleeding requiring at least 6 units of blood during 24 hours. Since the organ function might evolved over time, the time to determine organ failure outcome was set at 7 days after delivering ECMO treatment or censor time (death or bridging to heart transplantation or VAD) whichever came first.

### Data Collection

Patients’ basic demographics, pre-existing comorbidity, pre-ECMO CPR, peri-ECMO period procedure and use of inotropic agent, etiologies of circulatory deterioration, site of setting ECMO, outcomes and complications were prospectively collected and registered in an on-line data bank. To evaluate the initial severity of patients, the scoring systems including Acute Physiology and Chronic Health Evaluation II (APACHE II) score^24^, Sequential Organ Failure Assessment (SOFA) score^25^, Logistic Organ Dysfunction score (LODS)^26^, blood PH and lactic level, IE were recorded every day and were averaged for the initial 48 hours. To evaluate the changes of the patients’ conditions, laboratory data including serum lactic acid level, arterial blood gas, daily urine output amount, hemodynamic data including SBP and diastolic blood pressure (DBP), heart rate (HR), CVP and IE were recorded every day and the averaged value within one week after ECMO implantation were reported. LVEF was obtained by echocardiography every day and the averaged value within one week was reported.

### Propensity Score Methods

Due to small sample, we used inverse propensity score weighting (IPSW) to balance the observed variables in the two treatment groups^7^,^27^,^28^. The propensity score was the conditional probability of receiving IABP treatment, as a binary dependent variable, under a set of measurements. Clinical risk factors listed in Table 1 were added into a nonparsimonious multivariable logistic regression model to predict the probability of using IABP. The model included baseline characteristics (age, gender, body mass index, current smoker), pre-existing comorbidity (hypertension, diabetes mellitus, chronic kidney disease, dialysis therapy, liver cirrhosis, chronic obstructive pulmonary disease), cardiovascular disease (coronary artery disease, old myocardial infarction, stroke, peripheral artery disease), severity index (APACHEII, SOFA, LODS, PH, lactic acid, IE), pre-ECMO CPR, peri-ECMO period procedure, cause of ECMO treatment and ECMO set-up site. The predicted probability derived from the logistic equation was used as the propensity score for each individual. A subject’s weight was then defined as the inverse of the probability of receiving the treatment that the subject actually received. The weighting factor was used in the regression model.

### Statistical Analysis

The normality of the variables was tested by Shapiro-Wilk test. Continuous variables with normal distribution were expressed as mean ± standard deviation (SD), while those that were not normally distributed were reported as medians and quartiles (25% to 75%). Categorical variables were expressed as percentages. Continuous variables were compared by Student’s t –test for normal distributed variables and were compared by Mann-Whitney U test for non-normal distributed variables. Categorical variables were compared with Chi-Square test. To evaluate the effect of IABP usage to the short-term outcomes after ECMO treatment, logistic regression was performed to adjust potential confounders by adjusting for age, gender, pre-existing cardiovascular disease, CPR (yes or no), peri-ECMO operation (yes or no) and the severity index including APACHEII, SOFA and LODS. For IPSW, a binary generalized estimating equation (GEE) model was used to correct the “inflating” sample caused by weighting. The results were presented as odds ratio (OR) and 95% confidence interval (CI) and were stratified by etiologies of ECMO treatment. A *p* value less than 0.05 was considered statistically significant.

## Additional Information

**How to cite this article**: Lin, L.-Y. *et al.* Effects of Additional Intra-aortic Balloon Counter-Pulsation Therapy to Cardiogenic Shock Patients Supported by Extra-corporeal Membranous Oxygenation. *Sci. Rep.* **6**, 23838; doi: 10.1038/srep23838 (2016).

## Figures and Tables

**Table 1:** Basic characteristics of ECMO patients with and without IABP implantation.

	ECMO alone (N = 227)	IABP plus ECMO (N = 302)	p
**Baseline**			
Age	52.8 ± 17.2	56.8 ± 13.4	0.004
Gender, F, %	30.0	20.5	0.014
BMI, kg/m^2^	23.9 ± 4.3	25.1 ± 3.9	0.001
Smoker	20.4	28.5	0.034
**Pre-existing comorbidity, %**			
Hypertension	29.1	39.4	0.017
Diabetes mellitus	26.4	36.8	0.015
CKD	48.0	42.7	0.251
ESRD under dialysis	6.2	5.3	0.707
Liver cirrhosis	2.2	0.3	0.089
COPD	1.8	1.0	0.469
**Cardiovascular disease**			
CAD	22.7	24.2	0.402
Old MI	8.4	9.3	0.759
Stroke	5.3	8.6	0.174
PAD	2.2	1.7	0.751
**Initial severity index**			
APACHII	18.3 (11.6–23.4)	18.0 (12.3–23.3)	0.816
SOFA	14.0 (11.4–17.0)	13.4 (10.8–15.6)	0.254
LODS	9.1 (7.0–12.0)	9.1 (6.0–11.3)	0.958
PH	7.29 (7.23–7.32)	7.26 (7.20–7.32)	0.033
Lactic acid	4.6 (2.5–6.8)	4.5 (2.7–6.5)	0.826
IE	22.8 (13.6–40.6)	21.2 (12.0–38.7)	0.235
**Peri-ECMO period procedure, %**			
Pre-ECMO CPR	29.1	38.1	0.033
Peri-ECMO operation			
CABG	13.2	38.4	<0.001
Valvular surgery	11.9	4.0	0.001
Aortic surgery	1.3	0.0	0.078
Others	7.0	3.0	0.038
**Causes of ECMO, %**			<0.001
ACS	25.6	58.9	
Cardiomyopathy	27.3	17.2	
Post-cardiotomy	31.7	12.9	
Acute myocarditis	15.4	10.9	
**Set-up site**			<0.001
Ward	0.0	0.3	
Cath room	6.6	11.3	
Operation room	17.3	9.9	
Intensive care unit	41.6	28.8	
Emergency room	12.4	25.8	
Other hospital	15.0	20.5	
Others	7.1	3.3	

Abbreviations: ECMO, extracorporeal membrane oxygenation; IABP, intra-aortic balloon counter-pulsation; BMI, body mass index; CKD, chronic kidney disease; ESRD, end-stage renal disease. COPD, chronic obstructive pulmonary disease; CAD, coronary artery disease; MI, myocardial infarction; PAD, peripheral arterial disease; APACHEII, acute physiology and chronic health evaluation II; SOFA, sequential organ failure assessment; LODS, logistic organ dysfunction score; IE, inotropic equivalent; CPR, cardiopulmonary resuscitation; CABG, coronary artery bypass graft; ACS, acute coronary syndrome.

**Table 2:** Comparison of several blood and clinical parameters in patients with and without IABP implantation.

	ECMO (N = 227)	IABP plus ECMO (N = 302)	p
**Organ perfusion**			
Lactic acid	3.0 (2.0–4.4)	3.1 (2.1–4.6)	0.588
Urine output	913.0 (127.5–2388.9)	1332.9 (206.0–2190.6)	0.303
**Blood gas**			
FiO2	0.57 (0.48–0.69)	0.58 (0.50–0.67)	0.746
PH	7.21 (7.19–7.24)	7.21 (7.18–7.25)	0.807
PaO2	77.6 (58.7–103.7)	70.7 (54.7–103.3)	0.297
PaCO2	16.7 (14.3–23.3)	16.9 (14.6–21.4)	0.905
Bicarbonate	11.4 (10.2–13.4)	11.4 (10.1–13.3)	0.898
**Hemodynamics**			
SBP	99.5 (86.3–113.4)	107.6 (93.3–120.5)	<0.001
DBP	65.1 (54.4–73.1)	57.4 (50.3–64.8)	<0.001
HR	99.4 (88.1–112.3)	98.3 (87.5–107.5)	0.199
CVP	12.3 (10.0–14.9)	11.6 (9.9–14.0)	0.080
Inotropic equivalent	19.3 (10.4–33.4)	17.1 (10.1–29.1)	0.367
**Heart function**			
LVEF	30.0 (19.5–43.6)	30.0 (22.0–40.0)	0.946

Abbreviations: ECMO, extracorporeal membrane oxygenation; IABP, intra-aortic balloon counter-pulsation; FiO2, fraction of inspired oxygen; PaO2, partial pressure of oxygen in arterial blood; PaCO2, partial pressure of carbon dioxide in arterial blood; SBP, systolic blood pressure; DBP, diastolic blood pressure; HR, heart rate; CVP, central venous pressure; LVEF, left ventricular ejection fraction.

**Table 3:** Comparison of the incidences of different outcomes in patients with and without IABP implantation.

	ECMO (N = 227)	IABP plus ECMO (N = 302)	p
**ECMO duration, days**	4.0 (2.0–8.0)	4.0 (2.0–8.0)	0.964
**IABP timing**			
IABP initiation, hrs	N/A	0.0 (0.0–0.0)	N/A
IABP duration, days	N/A	5.0 (3.0–8.0)	N/A
**Organ failure**			
Brain	39.6	44.4	0.287
Lung	32.2	38.4	0.143
Heart	57.7	60.3	0.592
Liver	26.4	19.2	0.057
Kidney	48.9	52.3	0.482
Gastro-intestine	15.0	9.9	0.082
**Vascular complications**			
Need reperfusion	47.1	49.3	0.660
Fasciotomy	0.0	2.6	0.012
Digital gangrene	9.7	7.6	0.433
**Bridge to VAD or transplantation**			
VAD	0.9	0.0	0.184
Heart transplantation	7.5	4.0	0.085
**Two weeks mortality, %**	48.5	47.7	0.861
**Mortality etiologies**			0.830
MOF	92.2	94.2	
Brain death	2.8	3.2	
Major bleeding	1.4	1.1	
Others	3.5	1.6	

Abbreviations: ECMO, extracorporeal membrane oxygenation; IABP, intra-aortic balloon counter-pulsation; MOF, multi-organ failure (more than two organ dysfunction).

**Table 4:** Odds ratios for different outcomes (within 2 weeks after ECMO treatment) in patients with and without IABP implantation before (left) and after propensity adjustment (right).

	OR (95% C.I.) IABP plus ECMO vs. ECMO	P	OR (95% C.I.) IABP plus ECMO vs. ECMO	P
**ALL (N** **=** **529)**				
LVEF (>35 vs. ≦35%)	0.730 (0.473–1.126)	0.154	0.872 (0.526–1.447)	0.597
CVP (>15 vs. ≦15, cmH2O)	0.775 (0.457–1.313)	0.343	0.964 (0.531–1.749)	0.904
MOF	1.360 (0.810–2.282)	0.212	1.251 (0.716–2.188)	0.432
Vascular complications	1.008 (0.666–1.525)	0.955	1.018 (0.644–1.610)	0.939
Mortality	1.362 (0.801–2.314)	0.254	1.407 (0.760–2.604)	0.277
**ACS (N** **=** **236)**				
LVEF (>35 vs. ≦35%)	0.726 (0.330–1.597)	0.426	0.696 (0.302–1.603)	0.394
CVP (>15 vs. ≦15, cmH2O)	0.800 (0.249–2.568)	0.708	0.825 (0.204–3.328)	0.825
MOF	1.674 (0.630–4.445)	0.301	1.642 (0.613–4.396)	0.324
Vascular complications	0.812 (0.396–1.664)	0.570	0.883 (0.413–1.887)	0.748
Mortality	1.630 (0.575–4.622)	0.358	1.686 (0.585–4.859)	0.333
**Cardiomyopathy (N** **=** **111)**				
LVEF (>35 vs. ≦35%)	0.577 (0.160–2.085)	0.402	0.555 (0.147–2.102)	0.386
CVP (>15 vs. ≦15, cmH2O)	0.843 (0.289–2.462)	0.755	1.022 (0.350–2.983)	0.968
MOF	0.969 (0.314–2.991)	0.956	1.212 (0.394–3.732)	0.738
Vascular complications	0.895 (0.340–2.352)	0.821	0.978 (0.346–2.761)	0.966
Mortality	1.023 (0.298–3.513)	0.971	1.131 (0.294–4.351)	0.858
**Post-cardiotomy (N** **=** **114)**				
LVEF (>35 vs. ≦35%)	0.342 (0.102–1.147)	0.082	0.428 (0.100–1.836)	0.254
CVP (>15 vs. ≦15, cmH2O)	0.522 (0.131–2.078)	0.356	0.747 (0.150–3.720)	0.722
MOF	2.738 (0.914–8.206)	0.072	1.774 (0.539–5.835)	0.346
Vascular complications	1.206 (0.491–2.961)	0.683	1.158 (0.425–3.159)	0.774
Mortality	8.398 (1.916–36.805)	0.005	9.848 (1.523–63.672)	0.016
**Myocarditis (N** **=** **68)**				
LVEF (>35 vs. ≦35%)	0.407 (0.087–1.900)	0.253	0.873 (0.226–3.377)	0.844
CVP (>15 vs. ≦15, cmH2O)	5.004 (1.086–23.060)	0.039	3.424 (0.813–14.417)	0.093
MOF	3.179 (0.494–20.471)	0.224	3.895 (0.745–20.353)	0.107
Vascular complications	1.239 (0.285–5.378)	0.775	1.301 (0.316–5.360)	0.716
Mortality	3.059 (0.548–17.072)	0.202	3.654 (0.743–17.981)	0.111

Abbreviations: ECMO, extracorporeal membrane oxygenation; IABP, intra-aortic balloon counter-pulsation; LVEF, left ventricular ejection fraction; CVP, central venous pressure; MOF, multi-organ failure. Model adjusted for age, gender, pre-existing cardiovascular diseases, cardio-pulmonary resuscitation (yes or no), peri-ECMO operation (yes or no) and severity index (APACHEII, SOFA, LODS).
